# The Exciting Cause of Tetanus

**Published:** 1890-03

**Authors:** 


### The Exciting Cause of Tetanus.

The nature of tetanus and its cause have been of great interest to pathologists for many years. The experiments of Carle and Rattone, 1884, helped to clear up the mystery surrounding the disease. These observers were able to demonstrate that tetanus is an infectious and transmissible disease. In the following year Nicolaier found that there were bacilli in the superficial layers of the earth which, when inoculated in rabbits and guinea-pigs, were capable of producing the typical symptoms of tetanus, followed by fatal results. Soon after this, in 1886, Rosenbach showed that the bacilli of Nicolaier were also present in tetanus occurring in human beings. As, however, some observers failed to find them in the pus of patients with tetanus, and in animals the subject of experiment, these bacilli could not be regarded with certainty as the exciting cause of tetanus, especially since up to that time no one had succeeded in isolating the bacillus in pure cultures and in producing artificial tetanus by inoculation. Dr. Kitasato found the subject in this condition, and he appears to have accomplished what previous investigators in this field had failed to do.

The paper containing the results of his experiments was read before the last Congress of the German Surgical Society, and is published in full in the *Archiv fur klinische Chirurgie*, volume thirty-nine, part two. Kitassato asserts that the bacilli of tetanus can be isolated with certainty if the proper method is pursued. This consists in heating on a water bath, for half an hour or an hour, at a temperature of 176° Fahr., mixed (or impure) cultures which have previously been in an incubator for a few days; after being heated the cultures are transferred to closed vessels into which hydrogen is introduced. The whole culture is then maintained at a temperature of from 64° to 68° Fahr. In about a week the colonies began to form.

The bacilli of tetanus, as observed in the pure cultures, possess certain noteworthy characteristics. In the first place, they grow only where air is excluded. They can be cultivated upon weak alkaline agar-agar, and upon gelatine, and grow more rapidly in these media if from one-half to two per cent. of cane sugar is added to them. They give to the cultures an exceedingly offensive odor. They thrive best at a temperature of 97° to 100.5 Fahr. The bacilli form spores, when at the temperature named, in thirty-six hours. The spores are round, thicker than the bacilli, and are found at the end of the bacillus, so that the latter presents the bristle-like aspect described by Nicolaier. They are capable of pretty strong resistance to heat, are still capable of life after being heated for an hour at a temperature of 176° Fahr. in a moist condition ; on the other hand, if kept for five minutes at a temperature of 212° Fahr., in a steam apparatus, they are destroyed. The bacilli also exhibit a good deal of resistance to germicides. The most effective of the latter appears to be corrosive sublimate, in a solution of one part to one thousand of water, with one-half per cent. hydrochloric acid. In this solution the bacilli lose their virulence after half an hour.

Cultures containing spores which have been dried on silk threads and then kept some days in an exsiccator over sulphuric acid, and then in ordinary air, are still virulent after a lapse of several months ; spores mixed with sterilized earth are likewise virulent for a long time.

The bacilli of tetanus have a decided, but slight, peculiar, lively movement, which is increased when they are kept warm ; when spores are present, however, the bacilli are immovable. As regards staining, they take the aniline dyes, and also Gram's stain.

The specific character of these bacilli is shown by the fact that when mice are inoculated by the point of a platinum wire previously dipped in a fine culture, they become affected with typical tetanus without exception in twenty-four hours, and die after two or three days. Rats, guinea-pigs, and rabbits can also be infected if a somewhat larger quantity of the culture, according to the size of the animal, is employed.

Kitasato concludes, as the result of his experiments, that the presence of such foreign bodies as cotton, splinters of wood, etc., which formerly were thought to be necessary to obtain a successful result in inoculation, are not needed, the bacilli of tetanus being sufficient. It is disappointing, however, to read his frank admis-ion that the most careful search, in the case of animals dying of tetanus from inoculation, failed to find either spores or bacilli at the point of inoculation or in any of the organs. Moreover, he was unable to produce tetanus in other animals with the organs of an animal dying of tetanus, nor could he succeed in cultivating the bacilli from them. The explanation of this failure may be, as Kitasato suggests, that the bacilli very quickly disappear in the animal's body; but until this is proved there will be ground to dispute the specific character of Nicolaier's bacillus of tetanus. The whole tone of Kitasato's paper, however, is that of the true scientific investigator; he states what he has found and wherein he has failed, briefly and with no attempt at evasion or concealment. We sincerely hope that he will continue his investigations until the relation of Nicolaier's bacillus of tetanus, and also that of the chemical poison discovered by Brieger, will become perfectly free from all obscurity.

